# A Novel Protein Is Lower Expressed in Renal Cell Carcinoma

**DOI:** 10.3390/ijms15057398

**Published:** 2014-04-29

**Authors:** Ruili Guan, Yongde Xu, Hongen Lei, Zhezhu Gao, Zhongcheng Xin, Yinglu Guo

**Affiliations:** 1 Andrology Center, Peking University First Hospital, Peking University, Beijing 100034, China; E-Mails: guanruili@gmail.com (R.G.); xyongde@gmail.com (Y.X.); hongenlei@foxmail.com (H.L.); gaozhezhu@gmail.com (Z.G.); 2 Department of Urology, Peking University First Hospital and the Institute of Urology, Peking University, Beijing 100034, China; 3 National Urological Cancer Center, Beijing 100034, China

**Keywords:** engrailed-2, renal cell carcinoma, tissue biomarker

## Abstract

*Engrailed-2* (*EN2*) has been identified as a candidate oncogene in breast cancer and prostate cancer. It is usually recognized as a mainly nuclear staining in the cells. However, recent studies showed a cytoplasmic staining occurred in prostate cancer, bladder cancer and clear cell renal cell carcinoma. The inconsistency makes us confused. To clarify the localization and expression of EN2 in renal cell carcinoma, anti-EN2 antibody (ab28731) and anti-EN2 antibody (MAB2600) were used for immunohistochemistry (IHC) respectively. Interestingly, we found that EN2 detected by ab28731 was mainly presented in cytoplasm while EN2 detected by MAB2600 was mainly presented in nucleus. To further investigate the different patterns observed above, lysates from full-length *EN2* over expression in HEK293T cells were used to identify which antibody the EN2 molecule bound by western blot. Results showed ab28731 did not react with the lysates. For this reason, the novel specific protein detected by ab28731 was not the EN2 molecule and was named nonEN2. Then using the renal carcinoma tissue microarray and renal tissues, we found that the protein expression levels of nonEN2 in kidney tumor tissues was significantly lower than that in kidney normal tissues (*p* < 0.05), so was in renal cell lines. Taken together, nonEN2 is lower expressed and may play an important role in renal cell carcinoma.

## 1. Introduction

Renal cell carcinoma (RCC) is one of most common lethal malignancies and its incidence is increasing year by year with developed improved diagnosis [1]. Smoking [2], obesity [3], hypertension [4] and other factors [5] are usually considered as the causes of the developing renal cell carcinoma. In all RCC patients, clear cell renal cell carcinoma (CCRCC) accounts for approximately 70%–80%. Radical nephrectomy is the most effective method to cure the RCC patients but at the same time it may bring on some side effects such as hemorrhage, infection and other complications. Approximately 30% of cases have the risk of metastatic outcome after the operation and the survival time is just about 5–10 years. Pathogenic mechanism of RCC is still obscured until now. Over the last decade, many scientists have been focusing on developing early diagnostic markers, prognostic markers and therapeutic targets with different stages of occurrence in RCC; furthermore, some promising tissue biomarkers have also been reported in recent years. Carbonic anhydrase IX (CAIX) was thought to be the most significant molecular marker independently associated with poor survival in advanced RCC and also CAIX expression profiles in blood and tissue samples were correlated with histological subtypes of kidney cancer [6,7].

We are concentrating on the development of new specific biomarkers in RCC. EN2 was to be as the research object in our study in the light of its probable oncogenic role in breast cancer [8] and prostate cancer [9]. To our surprise, we obtained different immunolocalization patterns by using anti-EN2 antibody (ab28731, Abcam, Cambridge, MA, USA) and anti-EN2 antibody (MAB2600, R&D, Minneapolis, MN, USA) by IHC method. To confirm which antibody the real EN2 is corresponding to, antibodies were performed to react with the full-length EN2 overexpression lysates in 293T cells. Anti-EN2 antibody (ab28731) did not react with the lysates while the other EN2 antibody works well. NonEN2, specific bound to ab28731 was discovered and further was to investigate its expression and clinical significance on renal cell carcinoma in the following experiment study.

## 2. Results and Discussion

### 2.1. IHC (Immunohistochemistry) of NonEN2 Expression Using ab28731 Antibody

**Figure 1.**
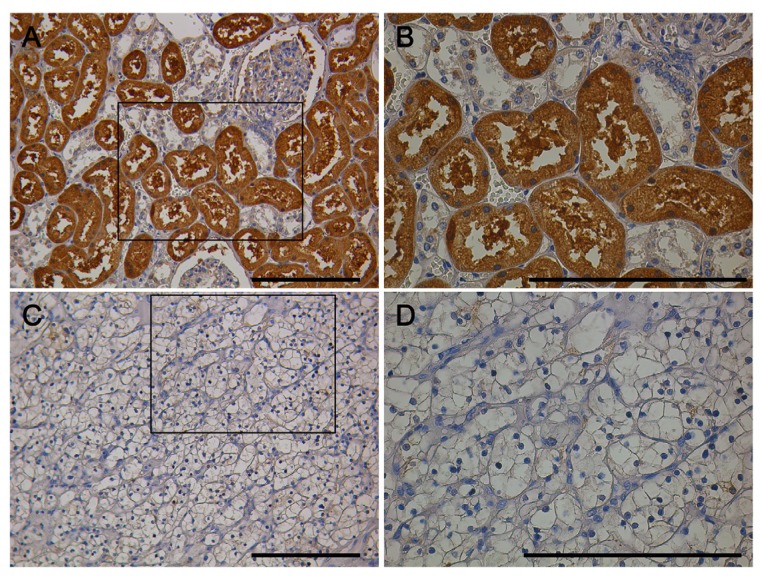
IHC (Immunohistochemistry) of nonEN2 expression using ab28731 in CCRCC (Clear Cell Renal Cell Carcinoma). (**A** and **B**) Kidney normal tissue, boxed areas in the 200× graphs are shown in the corresponding 400× graphs; (**C** and **D**) Kidney tumor tissue, boxed areas in the 200× graphs are shown in the corresponding 400× graphs. Insert bar = 100 μm.

Renal clear cell carcinoma tissue microarray (BC07114, Biomax, Rockville, MD, USA) containing 71 cases of kidney clear cell carcinoma (CCRCC), 13 kidney transitional cell carcinoma (TCC), 2 each of kidney carcinoma sarcomatodes (CS), papillary renal cell carcinoma (PRCC) and chromophobe carcinoma (CC), plus 10 normal kidney tissue (N), duplicate cores per case was performed for IHC by a polyclonal rabbit anti-EN2 antibody ab28731. IHC staining revealed that nonEN2 in kidney normal tissues and tumor tissues were mainly localized in the cytoplasm of kidney tubules and faintly in the nuclei (Figure 1).

### 2.2. IHC of EN2 Expression Using MAB2600 Antibody

**Figure 2.**
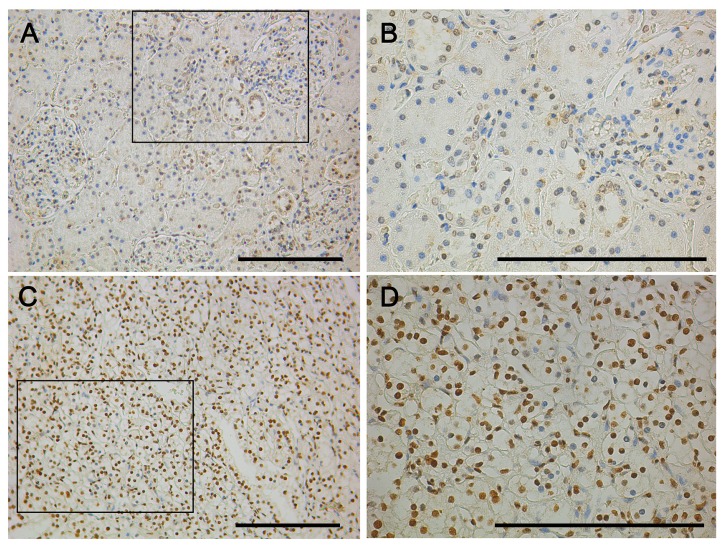
IHC of EN2 expression in CCRCC using MAB2600. (**A** and **B**) kidney normal tissue, boxed areas in the 200× graphs are shown in the corresponding 400× graphs; (**C** and **D**) kidney tumor tissue, boxed areas in the 200× graphs are shown in the corresponding 400× graphs. Insert bar = 100 μm.

A renal clear cell carcinoma tissue microarray BC07114 was performed for IHC by using a monoclonal mouse anti-EN2 antibody MAB2600 antibody. IHC staining suggested that EN2 was mainly localized in the nucleus and faintly in the cytoplasm (Figure 2). The result was on the contrary to that of ab28731.

### 2.3. Detection of Transient Overexpression Lysate of EN2 and Antibody Identification

**Figure 3.**
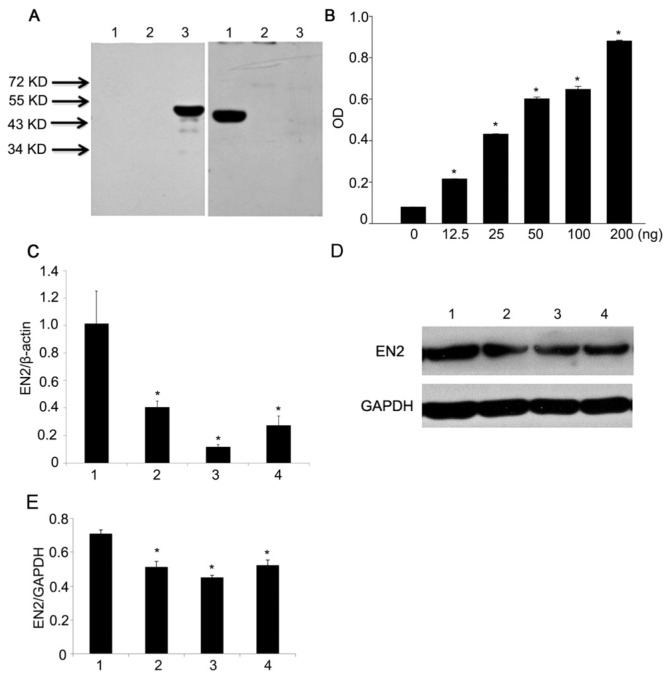
Western blot of EN2 overexpression lysates. (**A**) EN2 protein levels performed by ab45867 (left) and ab28731 (right) (lane 1: Lysates of HK2 cells, lane 2: Lysates of untransfected HEK 293T cells, lane 3: Lysates of EN2 overexpression in HEK 293T cells); (**B**) Indirect ELISA for peptide detection with the amounts of synthesized peptides increased with 2-fold change, * represents *p* < 0.05 when compared with the blank control. (**C**) qPCR analysis of lentiviral-mediated EN2 RNAi knockdown in 786-O cells (lane 1: Negative Control, lane 2: 1# siRNA, lane 3: 2# siRNA, lane 4: 3# siRNA); (**D**) Western blot analysis of lentiviral-mediated EN2 RNAi knockdown in 786-O cells (lane 1: Negative Control, lane 2: 1# siRNA, lane 3: 2# siRNA, lane 4: 3# siRNA); and (**E**) Semi-quantitative western blot analysis of EN2 RNAi knockdown. * represents *p* < 0.05 when compared with the lane 1.

HEK 293T cells in 6 well plate were transiently transfected with transfection Reagent and 2 μg DDK tagged-EN2 full-length cDNA plasmids. After 48 h, the cells were lysed in RIPA (KeyGEN Biotech, Nanjing, China) buffer and then centrifuged to clarify the lysate. Anti-DDK antibody were first used to confirm the success of transfection and overexpression by western blot (data not shown). Then full-length EN2 lysates produced by transient overexpression was used to verify which antibody the real EN2 was corresponding to. Results showed that MAB2600 and ab45867 reacted with the EN2 overexpression lysates well and they all presented a single bind at the same position (Figure 3A, left). However, ab28731 did not reacted with the EN2 overexpression lysates but could react with the lysates of HK2 cells (Figure 3A, right), suggesting that the protein blotted by ab28731 in HK2 cells was not EN2 molecule and named nonEN2 in this study.

The polyclonal antibody ab28731 was produced by immunizing the rabbits with synthesized peptides, derived from residues 284–333 (*C* terminal) of human EN2 according to the manuals from Abcam. In order to further confirm the affinity between peptides and ab28731, peptides (NESQIKIWFQNKRAKIKKATGNKNTLAVHLMAQGLYNHSTTAKEGKSDSE), which produced ab28731 were synthesized and coupled to a carrier protein KLH (Scilight Biotechnology, Beijing, China). Two batches of ab28731 from Abcam were tested to do the indirect ELISA. Results showed that the OD value significantly augmented as the amounts of synthesized peptides coated in the plate changed with 2-fold change. This means ab28731 reacted with the synthesized peptides derived from EN2 (284–333) (Figure 3B).

To further confirm the ab45867 antibody, a lentiviral-mediated EN2 RNAi knockdown was performed. Three siRNAs were transfected into 786-O cells for 72 h. qPCR was first done to make sure that success of EN2 gene knockdown (Figure 3C). Then, western blot was carried out to investigate the effect of EN2 protein reduction after EN2 RNAi knockdown (Figure 3D). The EN2 proteins levels after knockdown reduced significantly compared to the untreated cells when ab45867 was used to blot the cell lysates. The results demonstrated that ab45867 corresponds to the real EN2.

### 2.4. Comparison of EN2 Expression in Renal Cell Lines by Western Blot

**Figure 4.**
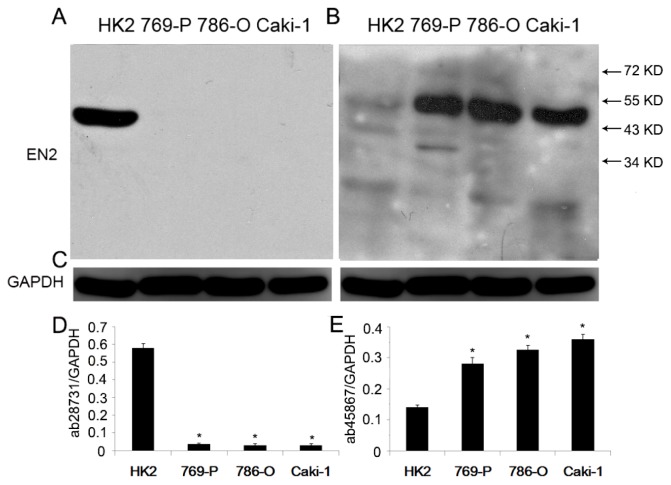
Western blot analysis of EN2 was performed on HK2 (lane 1), 769-P (lane 2), 786-O (lane 3), Caki-1 (lane 4). (**A**) Protein detected by using ab28731; (**B**) Protein detected by using ab45867; (**C**) GAPDH was used as an internal control to ensure equal loading. The same PVDF (polyvinylidene fluoride) membrane was used to react with the ab28731, ab45867 antibody followed by regeneration of the blot. Exposed films see the Supplementary Information; (**D** and **E**) A complete different expression patterns was observed by using the above two antibodies. * represents *p* < 0.05 when compared with the HK2 cells.

EN2 expression in renal cell lines was performed to use two antibodies including ab28731 and ab45867 by western blot. Proteins on PVDF (polyvinylidene fluoride) membrane were first used to react with ab28731 and followed ECL (electrochemiluminescence) explosion; the blot was incubated in stripping solution for 15 min at room temperature and re-blocked with 5% rabbit serum for 1 h, then ab45867 was used to react with the blot and followed ECL explosion. From Figure 4, protein levels were significantly decreased in renal cell lines by ab28731 compared to those in HK2 cells (Figure 4A,D). On the contrary, protein levels were remarkably increased in renal cell lines by ab45867 compared to those in HK2 cells (Figure 4B,E).

### 2.5. Western Blot of NonEN2 Expression in Renal Tissues

**Figure 5.**
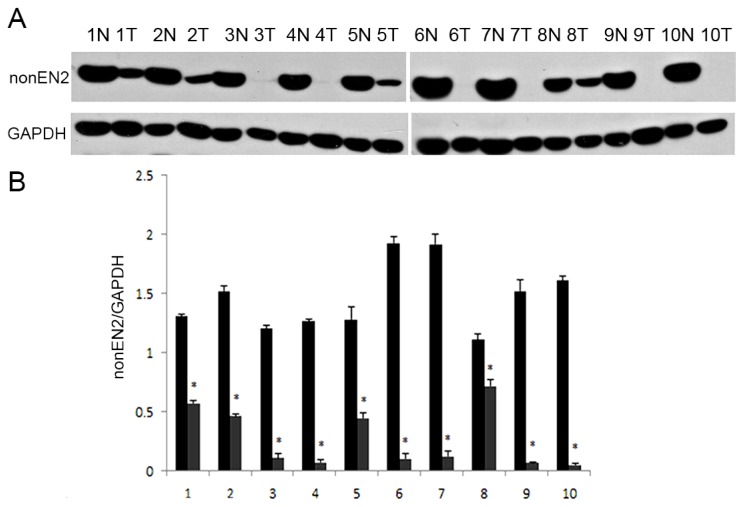
Western blot of nonEN2 protein in renal issues of CCRCC. (**A**) 10 paired primary tumor tissues (T), paired adjacent normal kidney tissues (N) specimens were done with western blot; (**B**) Semi-quantitative western blot data of relative proteins expression in renal tissues. * represents *p* < 0.05 when compared with the normal kidney tissues.

Ten paired primary tumor tissues (T) and adjacent normal kidney tissues (N) specimens were performed for western blot by using ab28731. From Figure 5, Protein levels of nonEN2 in kidney tumor tissues were sharply decreased compared with those in adjacent normal kidney tissues (*p* < 0.05).

### 2.6. IHC of NonEN2 Expression in Renal Cell Carcinoma

**Figure 6.**
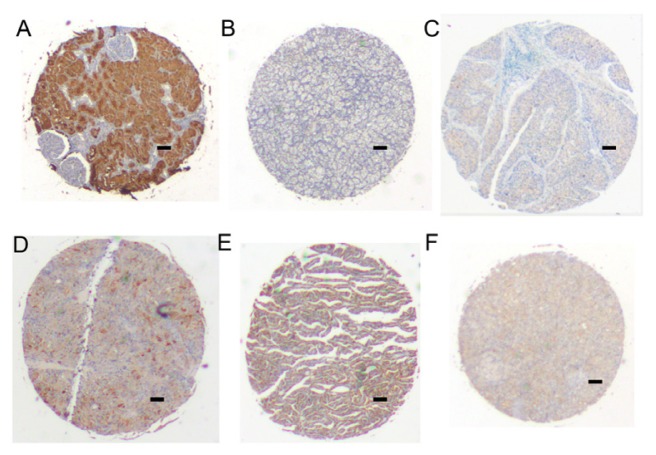
IHC of nonEN2 expression in renal cell carcinoma. (**A**) Normal kidney tissue; (**B**) Clear cell carcinoma; (**C**) Transitional cell carcinoma; (**D**) carcinoma sarcomatodes; (**E**) papillary renal cell carcinoma; and (**F**) chromophobe carcinoma. The magnification is 25× for the pictures photographed. Insert bar = 100μm.

Renal clear cell carcinoma tissue microarray BC07114 including 71 cases of kidney clear cell carcinoma (CCRCC), 13 kidney transitional cell carcinoma (TCC), 2 each of kidney carcinoma sarcomatodes (CS), papillary renal cell carcinoma (PRCC) and chromophobe carcinoma (CC), plus 10 normal kidney tissue (N), duplicate cores per case was performed for IHC by ab28731. The localization of nonEN2 was mainly presented in the cytoplasm and faintly in the nucleus staining whether in the normal tissues or other different types of renal cell carcinoma (Figure 6).

### 2.7. Semi-Quantitative Analysis of NonEN2 IHC in Renal Cell Carcinoma

**Figure 7.**
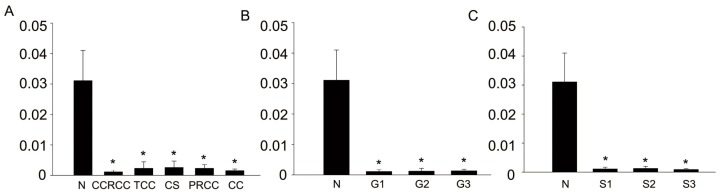
Semi-quantitative analysis of nonEN2 IHC in renal cell carcinoma. (**A**) Normal kidney tissue (N), clear cell renal carcinoma (CCRCC), kidney transitional cell carcinoma (TCC), carcinoma sarcomatodes (CS), papillary renal cell carcinoma (PRCC) and chromophobe carcinoma (CC); (**B**) Normal tissues (N), Grade 1 (G1), Grade 2 (G2) and Grade (G3) in CCRCC; and (**C**) Normal tissues (N), Grade 1 (S1), Grade 2 (S2) and Grade (S3) in CCRCC. * represents *p* < 0.05 when compared with the normal kidney tissues.

BC07114 was performed for IHC by ab28731. According to the pathological files, 71 cases of CCRCC include 44 cases of grade 1, 19 cases of grade 2 and 7 cases of grade 3; 71 cases of CCRCC include 53 cases of stage 1, 14 cases of stage 2 and 4 cases of stage 3. Then each spot was photographed at 200×. The staining was assessed by Image-Pro plus software (version 6.0, Media Cybernetics, Bethesda, MD, USA) and expressed as mean density. Data was analyzed by SPSS 17.0 statistical software program (version 13.0, SPSS, Chicago, IL, USA) to compare the difference between different groups. Each bar depicts the mean ± standard deviation. The results demonstrated that sharply decreased protein levels of nonEN2 were found in five types of renal cell carcinoma when comparing with those of normal tissues (*p* < 0.05) (Figure 7A). In addition, remarkably reduced nonEN2 expression occurs at any tumor grade or tumor stage in CCRCC compared with that in normal tissues, although there was no an association between different grades or different stages (Figure 7B,C).

### 2.8. Discussion

Renal cell carcinoma corresponds to 2% of new cases tumors of all adults in the world each year. The main histologic subtype of RCC is characterized as CCRCC [10]. The mechanism of pathology in CCRCC is obscure now and surgery is still an important and effective treatment for kidney patients. Biomarkers are urgent to be discovered and be used to predict the disease and monitor the prognostic conditions.

EN2 as a transcription factor was always recognized as oncogene in breast cancer and prostate cancer [8,9]. Recent studies reported that EN2 is a tumor specific urinary biomarker for the early diagnosis of prostate cancer and bladder cancer [11–13].

Previous study has confirmed that strong EN2 nuclear staining happened in breast cancer [8]. However, recent studies reported that a cytoplasmic staining was observed in prostate cancer (ab28731), bladder cancer (ab28731) and clear cell renal cell carcinoma [14]. The inconsistency makes us confused.

In addition, the role of EN2 expression in the genesis and development of RCC is still unclear. To understand the possible role of EN2 in RCC, we first used ab28731 and MAB2600 to investigate the EN2 expression by IHC. Surprisingly, two different localization patterns of EN2 expression were observed by using the above two antibodies. So which antibody is corresponding to the real EN2?

A DDK tagged-EN2 full-length overexpression system in HEK 293T cells was constructed to identify the antibodies. Antibodies including ab45867 and MAB2600 bound the overexpressed lysates well, while as ab28731 did not react with the lysates, revealing that a novel protein against 28731 but not EN2 molecule was discovered. Moreover, indirect ELISIA results also showed the 28731 could bind to the synthesized peptide, confirming that the polyclonal antibody ab28731 is absolutely produced by EN2 peptides (284–333), although it did not react with the EN2 molecule.

A renal cell microarray was used to investigate nonEN2 protein expression and localization. Strong cytoplasm staining and faint nucleus staining in the normal tissues and renal tumor tissues were observed by IHC. The expression of nonEN2 in kidney tumor tissues was sharply reduced or almost disappeared in RCC, especially in CCRCC. Western blot analysis also showed significant decreased nonEN2 expression in CCRCC compared with those in normal tissues. Combination the result of IHC and western blot, nonEN2 is a down-regulated protein candidate and may be involved in the development of RCC. Further studies are needed to investigate the new finding of nonEN2 molecule.

## 3. Experimental Section

### 3.1. Cell Culture and Tissues

All the tissues used in this study were collected from the urological department of the Peking University First Hospital with patients’ consents and hospital ethical committee approval. Renal cell lines 786-O, 769-P, Caki-1 and human kidney proximal tubular (HK2) cell lines were obtained from the ATCC (Rockefeller, MD, USA) and all cell lines were cultured according to ATCC protocols.

### 3.2. Immunohistochemistry (IHC)

Expression of EN2 in renal tumor tissue and normal tissue was performed on tissue microarray (BC07114, Biomax, Inc., Rockville, MD, USA). Anti-EN2 antibody (ab28731, Abcam) and Anti-EN2 antibody (MAB2600, R&D) diluted 1:100 were used to reacted with the microarray at 4 °C overnight after 3% H_2_O_2_ incubation for 10 min and antigen retrieval by heating at in microwave. A DAB system was used to detect the staining.

### 3.3. Western Blot

Cells and tissues was lysed on ice in RIPA (KeyGEN Biotech) buffers for 20 min and then centrifuged at 12,000× *g*, 15 min. Protein concentration was measured by BCA kit (Thermo Scientific, Pittsburgh, PA, USA). Proteins at 20 μg were loaded to 10% SDS-polyacrylamide gel electrophoresis and were transferred to a PVDF membrane (Millipore, Bedford, MA, USA). Anti-EN2 antibody (ab28731, Abcam), anti-EN2 antibody (MAB2600, R&D), anti-EN2 antibody (ab45867, Abcam), anti-GAPDH antibody and anti-DDK antibody (OriGene Technologies, Rockville, MD, USA) were used as the primary antibody for incubation at 4 °C overnight and the secondary antibodies labeled with horseradish peroxidase (HRP) as used for incubation at room temperature for 1 h. ECL chemiluminescent system was performed for the detection.

### 3.4. Transfection of EN2 into HEK293T Cells

Transient transfection was performed by using the plasmid DNA containing EN2 (NM_001427) Human cDNA ORF (open reading frame) Clone according to the protocol from Precision Shuttle TMVector System (OriGene Technologies, Rockville, MD, USA). In brief, HEK293T cells were seeded in a 6 well plate the day before transfection. 2 μg DNA was mixed with 1 μL LIPO2000 transfection Reagent (Invitrogen, Carlsbad, CA, USA) to form complexes at room temperature for 20 min. Then the complexes were added to each well and cultured at 37 °C for 48 h before collection. The cells were lysed in RIPA buffer and then centrifuged to clarify the lysate. Protein concentration was measured by BCA kit (Thermo Scientific). Cell lysates were stored at −80 °C before use.

### 3.5. Indirect ELISA for Peptide Detection

Peptides (NESQIKIWFQNKRAKIKKATGNKNTLAVHLMAQGLYNHSTTAKEGKSDSE) derived from residues 284–333 (*C* terminal) of human EN2, which produced anti-EN2 antibody (ab28731) were synthesized and coupled to a carrier protein KLH (Scilight Biotechnology, Beijing, China). Peptides (0, 12.5, 25, 50, 100, 200 ng) in carbonate buffer were coated in the wells of a PVC microtiter plate overnight and each has three replicate wells. Then, the plate was washed three times by filling the wells with 200 μL of phosphate-buffered saline (PBS). The remaining protein-binding sites in the wells were blocked by adding 200 μL of 1% BSA per well at 37 °C for 2 h, then incubated 1 h at room temperature and washed five times with 200 μL of PBS. The anti-EN2 antibody (ab28731, 1:1000) was added (100 μL) in each well and incubated for 1 h at 37 °C. The wells were then washed five times with 200 μL of PBS. The 100 μL HRP-secondary antibody (1:5000, ZSGB-BIO Company, Beijing, China) was added to each well and incubated for 30 min at 37 °C. Then, the plate was washed five times with 200 μL of PBS; 100 μL the TBM substrate was added to the wells and incubated at room temperature for 15 min in the dark. The reaction was then stopped with 100 μL of 0.3 M H_2_SO_4_ (stopping buffer) after sufficient color development. The absorbance of each well was read at 450 nm.

### 3.6. Lentivirus-Mediated EN2 RNAi Knockdown in 786-O Cells

The Lentiviral vector-mediated EN2 siRNA knockdown was performed to verify the ab45867 antibody. One nonspecific siRNA control (TTCTCCGAACGTGTCACGT) and three potential EN2 target sequences (1# AGTTCCAGACCAACAGGTA; 2# ACCCGAACAAAGAGGACAA; 3# GCAT CACCAACTTCTTCAT) were designed and done in this study (Genecheme, Shanghai, China). Pgcl-GFP vector was used to monitor the transfection effects. The MOI = 10 virus were transfected into the 786-O cells and then the cells were collected for the followed RNA and protein extraction after 72 h.

### 3.7. Quantitative Real-Time PCR (qPCR)

Total RNA from cultured cells was isolated using TRIZOL reagent (Invitrogen), and 2 μg total RNA was used to get the First-Strand cDNA Synthesis using the Reverse Transcription System (Cowin bioscience, Beijing, China). qPCR was performed using the Thermo Pikoreal PCR instrument with SYBR^®^ Green PCR Mix (Transgen biotech, Beijing, China). The PCR conditions consisted of 40 cycles, with 10 s denaturation at 94 °C, 30 s annealing at 60 °C, and 30 s for primer extension at 72 °C. EN2 and β-actin primers were synthetized as the previous report [11]. Target gene expression was normalized to the expression of the human actin for each sample. Data was analyzed using the 2^−ΔΔ^*^C^*^t^ method.

### 3.8. Statistical Analysis

For IHC, Image-pro plus software (version 6.0, Media Cybernetics) was used to calculate the mean density for each sample. Then semi-quantitative analysis was performed to compare the difference by SPSS 17.0 statistical software program (version 13.0, SPSS). One-way analysis of variance followed by a *post hoc* test was used to evaluate whether differences between groups were significant. *p* value < 0.05 was considered significant. For western blot, Image J software was used to measure band densities for each sample. All results are expressed as means ± SD. *p* value < 0.05 was considered significant.

## 4. Conclusions

Anti-EN2 antibody (ab28731), which is produced by EN2 peptide (284–333), did not react with the full-length EN2 protein but can react with the synthesized EN2 peptides. A novel protein nonEN2 was discovered in RCC and a sharply significant decrease occurs in RCC by IHC and western blot (*p* < 0.05), although nonEN2 expression levels were not associated with different clinical grades and stages in CCRCC. In conclusion, nonEN2 may play an important role in the development of RCC.

## Supplementary Information
