# Supplementary material for: A Novel Protein Is Lower Expressed in Renal Cell Carcinoma
Source: Int J Mol Sci. 2014 Apr 29;15(5):7398–408. doi: 10.3390/ijms15057398 (PMC4057679; doi:10.3390/ijms15057398)
Supplement: Supplementary file 1 [file ijms-15-07398-s001.pdf]

# Supplementary Information

**Figure S1.** Exposed films. A complete different expression patterns was observed by using the above two antibodies. (**A**) Protein detected by using ab28731; (**B**) Protein detected by using ab45867; (**C**) GAPDH was used as an internal control to ensure equal loading. The same PVDF (polyvinylidene fluoride) membrane was used to react with the ab28731, ab45867 antibody followed by regeneration of the blot; (**D** and **E**) A complete different expression patterns was observed by using the above two antibodies. * represents $p < 0.05$ when compared with the HK2 cells.

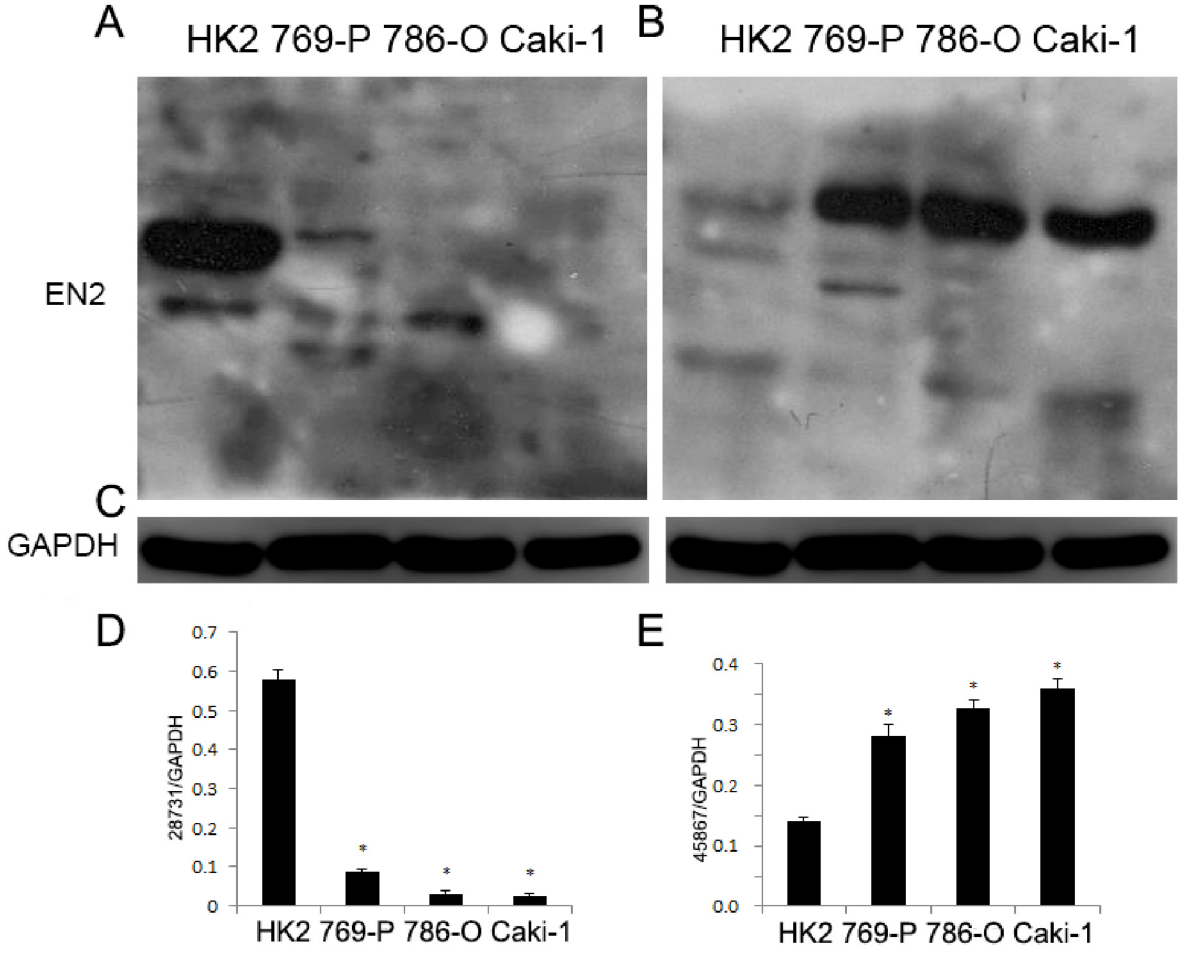


© 2014 by the authors; licensee MDPI, Basel, Switzerland. This article is an open access article distributed under the terms and conditions of the Creative Commons Attribution license (http://creativecommons.org/licenses/by/3.0/).
